# Early evaluation of optic nerve head morphology and choroidal thickness after PreserFlo MicroShunt implantation

**DOI:** 10.1007/s10792-022-02519-8

**Published:** 2022-09-21

**Authors:** Gloria Gambini, Matteo Mario Carlà, Tomaso Caporossi, Antonio Baldascino, Emanuele Crincoli, Umberto De Vico, Alfonso Savastano, Aldo Caporossi, Stanislao Rizzo

**Affiliations:** 1 Ophthalmology Department, Fondazione Policlinico Universitario A. Gemelli, IRCCS”, Largo A. Gemelli 8, 00168 Rome, Italy; 2 Catholic University “Sacro Cuore”, Rome, Italy

**Keywords:** Glaucoma, Preserflo Microshunt, Choroidal thickness, MIGS, Lamina cribrosa depth, Pre-laminar thickness

## Abstract

**Purpose:**

The aim of this study is to investigate changes in choroidal and optic nerve morphological parameters following MicroShunt PreserFlo implantation. The secondary aim is to investigate how the structural changes relate to the decrease in intraocular pressure (IOP).

**Methods:**

Prospective observational study on 15 eyes with glaucoma requiring MicroShunt implantation. Optical coherence tomography was used to measure macular choroidal thickness (MCT), peripapillary choroidal thickness (PCT), lamina cribrosa depth (LCD), cup depth and prelaminar tissue thickness (PLT), before and one day after surgery. Results were expressed in median and interquartile range (IQR) and correlated with IOP results.

**Results:**

The IOP decreased from a median of 25 (IQR = 11) mmHg to 8 (IQR = 2) mmHg the day after surgery. Median MCT increased after MicroShunt implantation from 252.1 (IQR = 156.4) µm to a postoperative value of 318.1 (IQR = 166.6) µm (*p* < 0.001), with a median increase of + 87.7 µm (+ 26.4%). PCT increased from 157.2 (IQR = 109.1) µm before surgery to 206.0 (IQR = 136.1) µm after surgery (*p* < 0.001). Moreover, we found a significant post-operative decrease in cup depth (median reduction of − 29.3 µm, *p* < 0.001) and an increase in PLT (median increase of 27.3 µm, *p* = 0.028). On the other side, LCD reduction 24 h after surgery didn’t reach any statistical significance.

**Conclusion:**

PreserFlo implantation determines retinal structural changes which appear similar to those caused by traditional filtering surgery, confirming the effectiveness of this device, meantime carrying a much smaller complications rate when compared to trabeculectomy.

## Introduction

The enhanced depth imaging optical coherence tomography (EDI-OCT) allows a higher image resolution of choroid and anterior surface of lamina cribrosa [1]. Hence, it has been used to investigate choroid and lamina cribrosa displacement following trabeculectomy and deep sclerectomy [2, 3].

Previous studies have shown an increase in macular and peripapillary choroidal thickness (CT), anteriorization of lamina cribrosa (LC) and an increase in the thickness of the prelaminar tissue following traditional glaucoma surgery. A positive correlation between the magnitude of change and the magnitude of IOP reduction has been found in some reports [4].

PreserFlo MicroShunt is a sub-Tenon filtering device which consists in a 8.5 mm tube composed of an inert polymer that induce minimal tissue fibrosis. The flow inside the 70-micron lumen of the device is laminar and there is no flow when the IOP inside the anterior chamber is less than 5 mmHg [5]. Several reports confirmed the effectiveness of this approach, with recent reviews gathering evidences regarding long-term IOP control and safety [6]. Furthermore, focusing on the early post-operative period, since this minimally invasive surgical technique doesn’t require viscoelastic substances to be left in the anterior chamber (AC), a better transparency of the dioptric elements is assured since the first post-operative follow up, allowing for high-quality imaging evaluation of device positioning and structural modifications.

To the best of our knowledge, the changes in choroidal thickness and in optic nerve head morphology following PreserFlo MicroShunt implantation have not been investigated yet. Moreover, previous reports of choroidal thickness and optic nerve head morphology changes, reported data from 7 days to 6 months after glaucoma surgery [3, 7]. The present study aims to investigate the earliest alteration of choroidal and optic nerve head structure analyzing EDI-OCT images acquired 24 h after surgery.

## Methods

This prospective observational study included glaucomatous patients who underwent MicroShunt PreserFlo implantation at Fondazione Policlinico Universitario A. Gemelli—Rome between January 27, 2021 and October 8, 2021.

The following inclusion criteria were considered: uncontrolled glaucoma on maximum tolerated medication or drug intolerance with an intraocular pressure (IOP) of 12–45 mmHg; phakic or pseudophakic patients; best-corrected visual acuity (BCVA) of 20/200 or better; and individuals with rapid and significant loss of visual function at visual field analysis [mean deviation (MD), pattern standard deviation (PSD), visual function index (VFI), and glaucoma progression analysis (GPA) and retinal nerve fiber layer (RNFL) analysis using Spectral Domain Optical Coherence Tomography (SD-OCT).

The exclusion criteria were: age under 18, history of intraocular inflammation or any retinal abnormalities, history of non-glaucomatous optic neuropathy, optic nerve drusen, low-quality image due to hyper-mature cataract, unstable fixation, refractive error more than – 6.0 or + 6.0 D of sphere and ± 3.0 D of cylinder. In case of concomitant cataracts, indications were given to perform PreserFlo implantation associated with phacoemulsification and IOL implantation. This study was conformed to the Declaration of Helsinki and informed written consent was obtained from all subjects.

Fifteen patients were included in this research and we collected parameters for both eyes, in order to compare treated eye with the fellow one.

### Examinations and measurements

Patients were clinically evaluated at baseline and 1 day after surgery. At baseline, all patients underwent a comprehensive ophthalmic examination including best-corrected visual acuity (BCVA), slit lamp examination, Tonopen (Reichert, NY, U.S.A.) and Goldmann applanation tonometry, gonioscopy, accurate fundus examination fundus with indirect ophthalmoscopy, i.e., an examination conducted with a slit lamp with a 90 D lens by Volk, corneal pachymetry (pachμmeter, Haag-Streit, Bern, Switzerland), computerized or manual visual field examination with SITA standard 30–2, Humphrey Field Analyzer—HFA II; Carl Zeiss Meditec, EDI-OCT of optic nerve head (ONH) and macula Spectralis^®^ SD-OCT Heidelberg Engineering GmbH, Heidelberg, Germany. In the case of concomitant cataracts, they underwent ocular biometry (IOLMaster 500; Carl Zeiss Meditec, La Jolla, CA, USA).

Follow-up examinations included: IOP, choroidal thickness in macula with a mean of 7 measurements at 500 μm distance, choroidal thickness in peripapillary area with a mean of 6 points at 500 μm distance, lamina cribrosa depth, CUP depth and prelaminar tissue thickness.

Morphological parameters of the optic nerve head and choroidal thickness of the treated eye were compared with the adelph eye, as the control, and related to IOP.

The choroidal layer and optic nerve head were imaged by SD-OCT EDI mode (Heidelberg Spectralis; Heidelberg Engineering, Dossenheim, Germany). All measurements were taken within a limited time frame from 8:00 am to 11:00 am to reduce the influence of diurnal variation of choroidal thickness. Images obtained were assessed by two ophthalmologists that were masked for patient status using caliper tool of Eye Explorer software version 5.4.

In each scan of ONH, a horizontal reference line was drawn connecting the two terminations of Bruch's membrane. Taking as a reference the deepest point of the cup, a vertical reference line was drawn. Two other vertical measurements were made at 100 μm and 200 μm from the vertical reference line. The distances from the horizontal reference line to the anterior surface of the prelaminar tissue were measured at the above three points. The average of the three measurements was defined as “CUP”. Distances from the same 3 points of the reference line to the anterior surface of the lamina cribrosa were also measured. The average of these measurements was defined as “lamina cribrosa depth” (LCD). The thickness of the prelaminar tissue (PLT) was considered as the difference between the cup and the LCD.

**Fig. 1:**
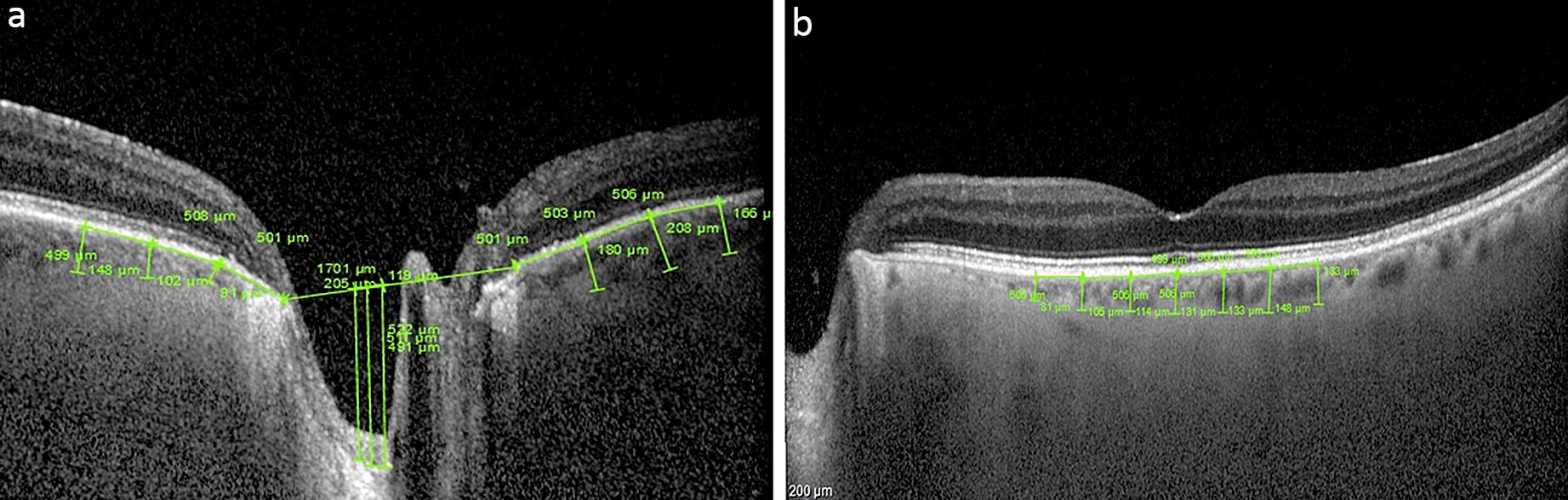
Optical coherence tomography (OCT) showing a schematic representations of optic nerve head and macular measurements. In each scan of ONH (**a**), a horizontal reference line was drawn connecting the two terminations of Bruch's membrane. Starting from the deepest point of the cup, a vertical reference line was drawn, and two other vertical measurements were drawn at 100 μm and 200 μm distance. The average distance of the three measurements from the horizontal line was defined as “cup”. Distances from the same 3 points of the reference line to the anterior surface of the lamina cribrosa were also measured, and their average defined the LCD. PLT was considered as the difference between the cup and the LCD. On peripapillary scans, the CT was manually segmented and measured between the Bruch membrane hyperreflective line and the hyperreflective line of the inner surface of the sclera, by 3 measurements spaced 500 μm for each sector, both nasally and temporally. In horizontal macular scans (**b**), CT was measured using the same anatomical boundaries. The subfoveolar CT was measured along with 3 measurements, equidistant 500 μm each from the fovea, in the nasal and temporal sectors, respectively. Mean macular CT was defined as the average of these seven measurements. *LCD* Lamina cribrosa depth; *PLT *Pre-laminary tissue; *CT* Choroidal thickness

On peripapillary scans, the choroid was manually segmented and measured between the Bruch membrane hyperreflective line and the hyperreflective line of the inner surface of the sclera. The CT was assessed in the temporal and nasal sectors by 3 measurements spaced 500 μm for each sector (Fig. 1).

In horizontal macular scans, CT was measured using the same anatomical boundaries. First, the subfoveolar choroidal thickness (SFCT) was measured and 3 measurements were then carried out equidistant 500 μm from the fovea in the nasal and temporal sectors, respectively.

Mean macular choroidal thickness (MCT) was defined as the average of these seven measurements (Fig. 1).

### Statistical analysis

The sample size calculation was performed using G*power (3.1.9.7 software) by setting the desired power of the study to 80%, the alpha error to 5% and a clinically significant difference of 7 µm in cup depth. Statistical analysis was conducted using SPSS software (IBM SPSS Statistics 26.0). Qualitative variables were described as number of cases over total and percentage. Since the distribution of quantitative variables resulted to differ from normality according to Shapiro–Wilk test, the analysis was performed with non-parametric statistics. Quantitative measures were thus described using median and interquartile range (IQR). Wilcoxon test was used to compare paired data for each group. Mann–Whitney test was performed to detect differences in the variation of the parameters (delta values) between the two groups (unpaired data). Linear correlation of quantitative values was assessed with Spearman test. The agreement between the two graders in manual measurements was determined through Bland Altman plot analysis. For all the aforementioned tests, a *p* value < 0.05 was considered as statistically significant.

## Results

Thirty eyes of 15 patients were included in the study. There were 7 men (46.7%) and 8 (53.3%) women. The average age of the patients treated at diagnosis was 67.3 years (range 45–85 years). All enrolled patients were Caucasian. In the study group, 14 eyes (93.2%) of 15 patients had a diagnosis of primary open angle glaucoma, 1 patient (6.7%) had a diagnosis of closed-angle glaucoma. Among patients diagnosed with open-angle glaucoma, 6 patients had a diagnosis of pseudoexfoliative glaucoma, 1 patient had a diagnosis of pigmentary glaucoma, 1 patient had a diagnosis of juvenile glaucoma and 7 patients had a diagnosis of primary open-angle glaucoma (POAG). At baseline, all patients were treated with at least 2 active principles. The mean of active topical drugs in use at the time of diagnosis was 3.7 ± 1.1. None of the eyes enrolled in the study had already undergone previous surgery involving the conjunctiva. Five eyes were pseudophakic at the time of Preseflo implantation. Out of the 10 phakic subjects who underwent PreserFlo implantation, 4 were simultaneously subjected to combined phacoemulsification and IOL implantation in association with PreserFlo. Manual measurements revealed sufficient closeness of agreement between graders for all the analyzed parameters. A review of demographic characteristics of the study population is available in Table 1.

**Table 1:** Descriptive analysis of the study population

Variable	Descriptive value
N° eyes	Total = 30 Treated = 15/30 (50%) Untreated = 15/30 (50%)
Male sex	8/15 (53.3%)
Age (years)	63 (14.5)
IOP at *T*0 (mmHg)	18.5 (8)
Type of glaucoma	POAG = 8/15 (53.3%) Pseudoexfoliative = 6/15 (40%) PACG = 1/15 (6.7%)
Pseudophakic	5/15 (33.3%)

*IOP *intraocular pressure, *POAG *primary open angle glaucoma, *PACG *primary angle closure glaucoma

In the study group, median preoperative IOP was 25 (IQR = 11) mmHg while median IOP at 1 day after surgery was 8 (IQR = 2) mmHg, indicating both a clinically and statistically meaningful variation induced by the surgery as a proof of its effectiveness (*p* < 0.001). Moreover, median decrease in IOP of − 13 (IQR = 10.5) mmHg in the treated eye differed significantly from median decrease of 0.0 (IQR = 2) mmHg detected in the fellow eye (*p* < 0.001). A review of all pre and post-operative morphological changes is available in Table 2.

**Table 2:** Results of paired analysis of OCT parameters and IOP before and after surgery for both treated and untreated eyes

Variable	Treated eye		*P*	Untreated eye		*p*
	Before surgery (IQR)	After surgery (IQR)		Before surgery (IQR)	After surgery (IQR)	
IOP (mmHg)	25 (11)	8 (2)	< 0.001	18 (3.5)	18 (3)	0.753
MCT (µm)	252.1 (156.4)	318.1 (166.6)	< 0.001	320.7 (178.2)	280.5 (168.8)	0.754
PCT (µm)	157.2 (109.1)	206.0 (136.1)	< 0.001	169.8 (113.4)	166.0 (110.3)	0.306
LCD (µm)	456.7 (186.5)	447.0 (157.6)	0.532	414.3 (164.3)	422.0 (179.5)	0.256
Cup (µm)	355.0 (187.2)	274.7 (203.8)	< 0.001	338.7 (255.7)	351.0 (247.5)	0.427
PLT (µm)	132.7 (174.5)	183.0 (177.5)	0.028	133.7 (104.7)	128.0(112.2)	0.191

Data are expressed in median value and IQR value (between brackets). P value in the 4th column refers to the statistical significance of the variations of the parameters of treated eyes before and after surgery. *p* value in the 7th column refers to the statistical significance of the variations of the parameters of untreated eyes before and after surgery

*IOP* intraocular pressure, *MCT* macular choroidal thickness, *PCT* peripapillary choroidal thickness, *LCD* lamina cribrosa depth, *PLT* prelaminar tissue thickness, *IQR* interquartile range

As concerns macular morphological parameters, MCT changed from a preoperative median value of 252.1 (IQR = 156.4) µm to a postoperative value of 318.1 (IQR = 166.6) µm in the treated eye (*p* < 0.001) while it didn’t change significantly in the fellow eye (see Table 2). As a confirmation to this, median increase in MCT after surgery was found to be + 87.7 (IQR = 34.3) µm in the treated eye compared to + 1.7 (IQR = 6.9) µm in the non-treated eye (*p* < 0.001). PCT also increased in the treated eye as a result of the surgery, passing from 157.2 (IQR = 109.1) µm before surgery to 206.0 (IQR = 136.1) µm after surgery (*p* < 0.001). Once again, this finding wasn’t present in the control group (*p* = 0.306). Table 3 summarizes the variations of clinical and morphological parameters in comparison to the untreated eye.

**Table 3:** Comparison of the variations of IOP and OCT parameters before and after surgery in treated and untreated eyes (unpaired data)

Variable	Treated eye (IQR)	Untreated eye (IQR)	*p*
ΔIOP	− 13 (10.5)	0.0 (2)	< 0.001
ΔMCT	87.7 (34.3)	1.7 (6.9)	< 0.001
ΔPCT	44.2 (32.8)	− 3.2 (8.1)	< 0.001
ΔLCD	− 16.3 (71.2)	3.7 (9.2)	0.547
ΔCup	− 29.3 (48.9)	− 5.0 (18.3)	< 0.001
ΔPLT	27.3 (69.6)	2.7 (12.5)	0.141

Data are expressed in median value and IQR value (between brackets)

Δ = difference from pre-operative and post-operative value

*IOP* intraocular pressure, *MCT* macular choroidal thickness, *PCT* peripapillary choroidal thickness, *LCD* lamina cribrosa depth, *PLT* prelaminar tissue thickness, *IQR* interquartile range

Comparing the nasal sector with the temporal sector, PCT increase was, respectively, + 25.17% and + 36.6%. However, this difference was not statistically significant (*p* = 0.12). No significant correlation was found between the magnitude of increase in macular and peripapillary CT and the magnitude of reduction of the IOP after surgery. Also no correlation was found between IOP at baseline and changes in choroidal structural parameters after surgery.

**Fig. 2:**
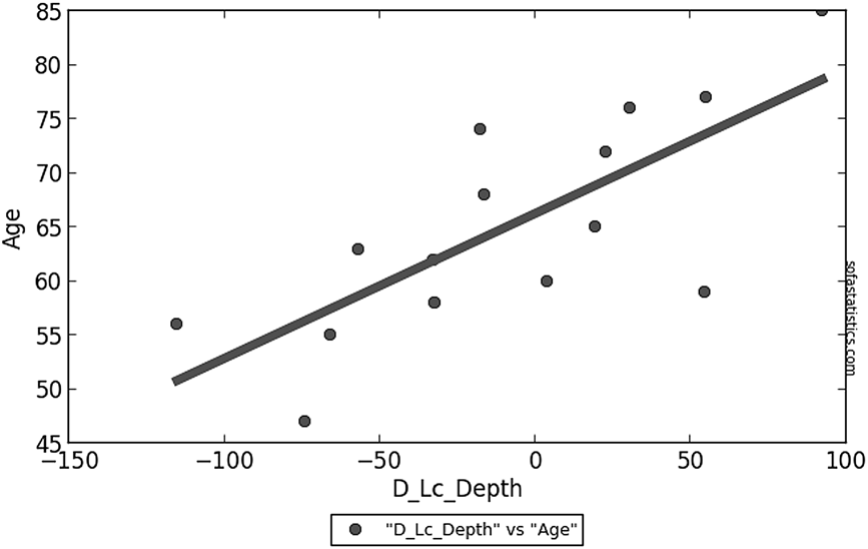
Linear correlation between age and variation of lamina cribrosa depth

Median preoperative and postoperative LCD in the study group were 456.7 (IQR = 186.5) µm and 447.0 (IQR = 157.6) µm, respectively, a reduction that didn’t reach statistical significance. Despite the detection of a median decrease in LCD in the treated eye of − 16.3 (IQR = 71.2) µm compared to an increase of + 3.7 (IQR = 9.2) µm in the control eye, also this difference wasn’t found to be significant. Nevertheless, we detected a liner correlation between the age of the patient and LCD variation in the treated eye following surgery (P value < 0.001, R = 0.761, Slope = 0.134, Intercept = 66.3) (visible in Fig. 2), a finding which was not present in the fellow eye.

By contrast, cup depth in the study eye varied significantly comparing pre and postsurgical measurements (respectively 355.0 (IQR = 187.2) µm and 274.7 (IQR = 203.8) µm, *p* < 0.001) and median surgical induced variation differed significantly from the control eye (respectively − 29.3 (IQR = 48.9) µm and − 5.0 (IQR = 18.3) µm, *p* < 0.001). In addition, PLT measurements revealed a significant increase after surgery in the study eye (from 132.7 (IQR = 174.5) µm preoperatively to 183.0 (IQR = 177.5) µm postoperatively, *p* = 0.028) while the contralateral eye’s increase didn’t reach statistical or clinical significance. Nevertheless, comparison between the groups as concerns pre and postoperative PLT variation didn’t detect any statistically significant difference between the two (*p* = 0.141). In order to evaluate the secondary objective, the IOP and the structural parameters taken into consideration were correlated. No particular correlations emerged from this analysis.

## Discussion

In accordance with previous studies performed on trabeculectomy, we found a significant increase in CT both in macula and in peripapillary region after the decline of the IOP, already 24 h after surgery [3, 7–10].

In literature, the increase of CT after filtering surgery has been attributed to different factors including the increase in the size and number of fenestrations of the capillaries at the level of choriocapillary and the active synthesis of osmotically active proteoglycans that attract fluid at the level of the choroid. This mechanism finds confirmation in the study carried out by Zhang et al. [11] in which an increase in postoperative thickness of both the intravasal component and the interstitio emerges.

At time T1, we also found a significant increase in the thickness of the prelaminar tissue with a consequent reduction of the cup. This data, even with a shorter follow up, agrees with the findings published by Bouillot et al., who observed a significant increase in the thickness of the prelaminar tissue at 1 week after surgery both in the trabeculectomy and in the deep sclerectomy group. The same data have been reported by Lee et al., up to 6 months after filtering surgery and by Agoumi et al. and Fazio et al., who observed the inverse phenomenon, i.e., a reduction of prelaminar tissue thickness after sudden increase in IOP [12–15.

Many explanations are reported in the literature about the mechanism underlying this phenomenon. Previous studies hypothesized that the increase in thickness is mainly related to an increase in the size of the vessels due to the increase in perfusion after the reduction of the IOP [16]. Reis et al. [17] suggest, however, that this increase is secondary to a change in the size of axons and astrocytes given the recovery of a greater axoplasmic flow. The findings from out study, collected at the first 24 h after surgery, may suggest that these mechanism occurs earlier than previously described in literature.

With regard to the LCD, we found an anteriorization of the lamina cribrosa which was not statistically significant 24 h after MicroShunt implantation. Our data analyzed the immediate morphological alterations of the LC after the filter surgery, differently from other studies in literature, which evaluated this parameter at 1 week, 1 month, 3 months and 6 months after surgery [3, 10]. Compared to prelaminar tissue, the lamina cribrosa has an immediate elastic response to pressure gradients and has also late plastic capacities of secondary adaptation to remodeling. We can, therefore, conclude that the anteriorization of the lamina cribrosa that other authors found from 1 week to 6 months after traditional surgery is mainly attributable to the plastic remodeling mechanisms that probably cannot yet be triggered only 24 h after surgery.

In our study, the magnitude of morphological changes in the choroid and laminar region did not appear to be correlated with the IOP reduction magnitude or the baseline IOP value. Conversely, Buillot et al. found a correlation between IOP variation linked to the MicroShunt and changes in macular choroidal thickness one week after surgery [7].

With the exception of few researches in which a relationship between the IOP and structural changes was not found, since the measurements were influenced by circadian variations, the majority of data published in literature show a significant correlation between the percentage of IOP reduction and the percentage of modification of structural parameters following traditional filtering surgery [3]. We hypothesized that the lack of correlation between the decline of IOP and the changes of structural parameters evaluated in our work may be related to the different surgical techniques used. In the case of MicroShunt implant, the surgical technique involves minimal manipulation of tissues with reduced operating time and the absence of manipulation of the anterior chamber, as also demonstrated by the lack of need to use viscoelastic to maintain the volumes in the postoperative period. Almost for the entire duration of the surgery, the technique takes place with the bulb closed up till the moment of implantation of MicroShunt which guarantees a uniform flow controlled by Poiseuille's law. Therefore, we thought that the absence of hypotonia and IOP fluctuations during and after surgery may possibly explain the lack of correlation between delta IOP and changes in structural parameters.

Furthermore, in patients with advanced glaucomatous damage treated with traditional filtering surgery, the loss of central vision known as "wipe-out" syndrome has been described [17–19]. This syndrome appears to be related to intraoperative and postoperative hypotonia that occurs after traditional surgery and seemed to be at more risk patients with higher IOP at baseline [20]. It has been suggested that the pathophysiological mechanism that explains the aforementioned phenomenon is similar to the sudden reperfusion after prolonged ischemia that occurs in crush injuries. This acute reperfusion results in the release of free radicals and endothelial damage. The sudden reduction in IOP and consequent decompression of the axons could similarly explain the cell death that occurs during "wipe-out" syndromes [20]. In our study, no cases of “wipe-out” syndromes were described, suggesting that this minimally invasive procedure does not determine a sudden and abrupt reduction in IOP, thus not inducing destructing changes to structural parameters. We, therefore, believe that there is a gradual decompression on the ocular structures facilitating the establishment of compensation mechanisms, making this a further element of confirmation of the increased safety profile that the MicroShunt presents when compared with traditional surgery. Due to these considerations, the absence of correlation between IOP percentage decrease and the magnitude of modifications to structural parameters should therefore be considered as an advantage of this procedure.

One of the main limitations of the study presented is that the series includes a high percentage of patients with pseudoexfoliative glaucoma. This condition affects the biomechanical properties of elastic tissues, including the lamina cribrosa. Vazquez et al. have shown that patients with this condition have greater weakness of lamina cribrosa, and consequently are more likely to undergo structural alterations following IOP changes after surgical procedures [21].

Further limitations of the present study are: the sample size, the lack of an arm undergoing late filtering surgery to be able to directly compare the parameters evaluated, the lack of complete data of the long-term follow-up, currently in progress, which allows for the evaluation about how these parameters may change over time.

This study is the first to investigate early structural changes after MicroShunt PreserFlo implantation. Thanks to the minimally invasive nature of the procedure, it was possible to perform high quality EDI-OCT scans already 24 h after surgery. In agreement with the data presented in literature focusing on retinal structural changes after filtering surgeries, we found a statistically significant increase in macular and peripapillary choroidal thickness and prelaminary tissue thickness. The magnitude of modification of structural parameters was not found to be related to the magnitude of reduction of the IOP. This finding, in association with the possibility of early evaluation of these parameters, represents a further index of safety and minimally invasive procedure.

We speculate, therefore, that the PreserFlo MicroShunt implantation is a minimally invasive technique compared to traditional filtering surgery, not only at the surgical site level, but also at the level of the structures functionally involved in the progression of glaucomatous opticopathy.
